# Urinary Tract Infections in the First 6 Months after Renal Transplantation

**DOI:** 10.1155/2021/3033276

**Published:** 2021-11-15

**Authors:** Ziad Arabi, Khalefa Al Thiab, Abdulrahman Altheaby, Ghaleb Aboalsamh, Samy Kashkoush, Mohamad Almarastani, Mohammed F. Shaheen, Abdulrahman Altamimi, Wael O'hali, Khalid Bin Saad, Lina Alnajjar, Rawan Alhussein, Raghad Almuhiteb, Bashayr Alqahtani, Rayana Alotaibi, Marah Alqahtani, Mohammed Tawhari

**Affiliations:** ^1^Division of Adult Transplant Nephrology, King Abdulaziz Medical City, Riyadh, Saudi Arabia; ^2^King Abdullah International Medical Research Center, Riyadh, Saudi Arabia; ^3^College of Medicine, King Saud Bin Abdulaziz University for Health Sciences, Riyadh, Saudi Arabia; ^4^Pharmaceutical Care Department, King Abdulaziz Medical City, Riyadh, Saudi Arabia; ^5^Department of Hepatobiliary Sciences and the Organ Transplant Center, King Abdulaziz Medical City, Riyadh, Saudi Arabia; ^6^Department of Pharmacy Practice, College of Pharmacy, Princess Nourah Bint Abdulrahman University, Riyadh, Saudi Arabia; ^7^College of Pharmacy, Princess Nourah Bint Abdulrahman University, Riyadh, Saudi Arabia

## Abstract

**Purpose:**

Urinary tract infections (UTIs) are common in the first 6 months after renal transplantation, and there are only limited data about UTIs after transplantation in Saudi Arabia in general.

**Methods:**

A retrospective study from January 2017 to May 2020 with 6-month follow-up.

**Results:**

279 renal transplant recipients were included. Mean age was 43.4 ± 16.0 years, and114 (40.9%) were women. Urinary stents were inserted routinely during transplantation and were removed 35.3 ± 28 days postoperatively. Ninety-seven patients (35%) developed urinary tract infections (UTIs) in the first six months after renal transplantation. Of those who developed the first episode of UTI, the recurrence rates were 57%, 27%, and 14% for having one, two, or three recurrences, respectively. Late urinary stent removals, defined as more than 21 days postoperatively, tended to have more UTIs (OR: 1.43, P: 0.259, CI: 0.76–2.66). Age >40, female gender, history of neurogenic bladder, and transplantation abroad were statistically significant factors associated with UTIs and recurrence. Diabetes, level of immunosuppression, deceased donor renal transplantation, pretransplant residual urine volume, or history of vesicoureteral reflux (VUR) was not associated with a higher incidence of UTIs. UTIs were asymptomatic in 60% but complicated with bacteremia in 6% of the cases. Multidrug resistant organisms (MDROs) were the causative organisms in 42% of cases, and in-hospital treatment was required in about 50% of cases. Norfloxacin + Bactrim DD (160/800 mg) every other day was not associated with the lower risk of developing UTIs compared to the standard prophylaxis daily Bactrim SS (80/400 mg).

**Conclusion:**

UTIs and recurrence are common in the first 6 months after renal transplantation. Age >40, female gender, neurogenic bladder, and transplantation abroad are associated with the increased risk of UTIs and recurrence. MDROs are common causative organisms, and hospitalization is frequently required. Dual prophylactic antibiotics did not seem to be advantageous over the standard daily Bactrim.

## 1. Background

Urinary tract infections (UTIs) are common among kidney transplant recipients in the first year after transplantation. The reported incidence varies from 11.7% to 67.5% based on the definition and the design of studies [1, 2]. UTIs remain a leading cause of hospitalization after kidney transplantation [3].

Several risk factors were identified and are thought to increase the risk of UTIs after renal transplant. These include older age, female gender, diabetes, history of acute rejection [2], delayed graft function [4], deceased donor kidney transplantation, longer duration of dialysis [5], and urological abnormalities [3]. Timing of stent removal and the use of antibiotic prophylaxis are important modifying factors. In this study, we retrospectively review the data from our transplant center pertaining to the development of UTIs in the first 6 months after renal transplantation. There are only limited data about UTIs after transplantation in Saudi Arabia in general [6–8].

## 2. Methods

After obtaining the institutional board review approval (RC20/138/R), a retrospective study was conducted to review the charts of renal transplant recipients at King Abdulaziz Medical City, Riyadh, Saudi Arabia, from January 2017 to May 2020 with 6-month follow-up.

Demographic patients' data, comorbidities, and renal transplantation data were collected. Transplant outcomes and complications with specific focus on posttransplant UTIs were collected. These included the incidence, potential risk factors, symptomatology, prevalence of multidrug-resistant organisms (MDROs), need for hospitalization, and treatment.

We classified UTIs similar to the previous studies [9–11] according to their symptoms as follows:

1. Asymptomatic bacteriuria: >10^5^ colony-forming unit (cfu)/mL
2. Simple (uncomplicated) UTI: positive urine culture in addition to any urinary symptoms such as dysuria, urgency, frequency, or suprapubic pain
3. Complicated UTI: positive urine culture in addition to systemic symptoms such as fever, chills, and flank/allograft pain
4. Complicated UTI with bacteremia
5. Recurrent UTI: more than one UTI in the first 6 months

### 2.1. Statistical Analysis

All analyses were performed using IBM SPSS software 23.0 (IBM Co., Armonk, NY, USA). Continuous variables were presented as means ± standard deviation (SD). Categorical variables were expressed as numbers and percentages. We compared data using the *t*-test, Mann–Whitney *U* test, chi-squared, or Fisher's exact tests as appropriate. Multivariable risk factor analysis was performed using logistic regression analysis. All statistical tests were two-sided, and *P* values <0.05 were considered statistically significant.

## 3. Results

A total of 279 renal transplant recipients were included. The mean age was 43.4 ± 16.0 years; 114 (40.9%) were women. Eighty percent of the participants received living donor transplant. Ninety-seven patients (35%) developed UTI in their first six month after renal transplantation (Table 1).

The first UTI occurred in 40.8 ± 44.5 days from transplant. For those who developed first UTI, the recurrence rate was 57%, 27%, and 14% for the first, second, and third recurrences occurring in 70.1 ± 45.4, 89.9 ± 39.9, and 124.2 ± 44.5 days from transplant, respectively (Table 2).

Most of the UTIs occurred in the first 2 months. Recurrence was mostly in the second month of transplant, and it decreased with time. UTIs were asymptomatic, simple, complicated, or complicated with bacteremia in 63.7%, 14.4%, 12.4%, or 6.2%, respectively.

### 3.1. Factors

Age >40 (P: 0.012, OR: 2.176, CI: 1.187–3.986), being a female (*P* < 0.001, OR: 5.008, CI: 2.74–9.156), receiving renal transplant abroad (*P* < 0.001, OR: 5.008, CI: 2.607–27.05), and being diagnosed with neurogenic bladder (*P* : 0.048, OR:5.646; CI:1.016–31.379) were important factors associated with UTIs and recurrences, whereas diabetes, the type of transplant (deceased donor versus living donor), pretransplant residual urine volume, and the presence of vesicoureteral reflux (VUR) were not associated with the higher incidence of UTIs. Similarly, urinary leak or ureteral stenosis was not associated with the increased incidence of UTIs. In addition, there was no association between the renal function (i.e., serum creatinine at 1 and 6 months after transplantation) and UTIs.

The level of immunosuppression (induction type, episodes of acute rejection, or detection of polyoma viremia) was not associated with the higher incidence of UTI.

In our study, the urinary stents were cystoscopically removed at a mean of 35.3 ± 28 days postoperatively. When the urinary stent was removed after 21 days, there was a trend towards a higher incidence of UTIs although it was not statistically significant (OR 1.43, P: 0.259, CI: 0.76–2.66). Of note, when stents were removed urgently (for non-UTI reasons such migrated or fallen stents), there were less UTIs.

Dual antibiotic prophylaxis (norfloxacin along with Bactrim DD) when compared to the standard prophylaxis with Bactrim SS daily alone was not associated with the decreased risk of UTIs (Table 3).

UTIs were asymptomatic in about 60% or complicated with bacteremia in 6% of the cases. Multidrug-resistance organisms (MDROs) were the causative organisms in 42% of cases. In-hospital treatment was required in about 50% (Table 2).

## 4. Discussion

**Figure 1:**
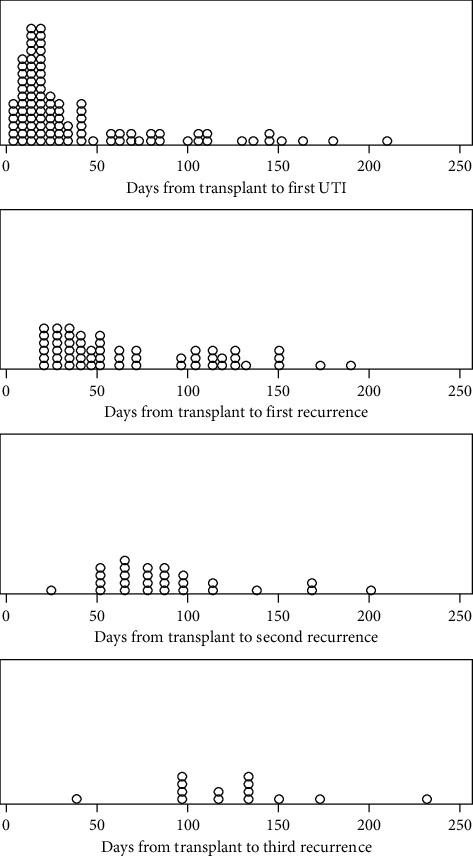
Timing of UTIs and recurrence.

In our study, the incidence of UTIs in the first 6 months after renal transplantation was 35%. Most of the UTIs occurred in the first 2 months, and recurrence was mostly in the second month of transplant and it decreased with time.  Figure 1 shows the timing of UTI and its recurrence. Although this incidence is high, it remains comparable and follows a similar course to what is reported in other studies [1, 2, 9, 12].

At our center, we stop routine screening with urine cultures beyond 6 months. This is due to the lack of evidence that treatment of asymptomatic UTIs has an impact on patient and graft outcomes including hospitalization [13, 14]. That is true, especially if the urinary stent has already been removed.

In our study, age >40, female gender, transplantation abroad, and history of neurogenic bladder were identified as important factors of UTIs and recurrence, whereas diabetes and the type of transplant (deceased versus living donor) were not. These findings are in line with previously published studies [12, 15–19].

Urological abnormalities are important factors for UTIs after renal transplantation [3]. In our study, neurogenic bladder and intermittent urinary self-catherization but not the presence of vesicoureteral reflux (VUR) were associated with the higher incidence of UTIs. This may be due to the low number of people with VUR (2.5%) and can also be related to the absence of the VUR in the transplanted kidney [20].

Our induction protocol consisted of basiliximab for low-immunological-risk transplantation and thymoglobulin (rATG) for high-immunological-risk transplantation. We use the standard triple immunosuppression therapy (tacrolimus, mycophenolate, and prednisolone) [21]. The association between the type of induction agent and UTIs has not been consistent [12, 15]. Our study does not show an association between the induction agent and post-renal-transplant UTIs. Our report also found no association between acute rejection and UTIs [12, 18].

No observed association was noted between the renal function after transplantation and the development of UTIs. This finding is in keeping with some previously published reports as well. For example, Papasotiriou et al. found no association between graft function and UTI [12]. Other researchers, however, found that the occurrence of complicated UTI has a negative impact on graft function [22].

Similarly, the incidence of urinary leak or ureteral obstruction after transplantation was low in our study and was not associated with a higher incidence of UTIs. Pretransplant residual urine volume was not a risk factor for UTI either.

Unlike our findings, some studies showed that diabetic nephropathy as a cause of ESRD is a risk factor for recurrent UTIs [23].

Shorter duration of effective therapy has been implicated as a risk factor for recurrent UTIs [23]. Our approach in regard to the duration of antibiotic treatment depends on the symptoms and complexity of the UTIs [24]. We treat asymptomatic UTI in the first 6 months after transplantation, especially if the urinary stent is still in place. We also treat simple UTIs for one week. However, we treat more complex UTIs (especially MDRO) for more prolonged durations (two weeks or at least one week plus subsequent suppressive antibiotic therapy such as nitrofurantoin or fosfomycin in the case of susceptible organism). Centers may follow slightly different recommendations for the management of UTI in kidney transplantation [25]. Patients who were transplanted abroad seemed to have a much higher risk for all complications including UTIs and recurrence of UTIs. This could be related to the difference in surgical techniques and standards of transplantation practices as this may happen under less-than-ideal precautions such as in the case of transplant tourism.

Ureteric stents may help to reduce early postoperative complications (leak and stricture) but increase the likelihood of UTI [26]. Infection while having a ureteric stent was associated with a high recurrence rate of UTI even after stent removal [27]. Studies have showed that earlier stent removal (<3 weeks) may decrease the incidence of UTIs without increasing the incidence of urinary leakage [28].

Our center used to target removal of the stent in the first 8 weeks after living donor kidney transplantation and 12 weeks after deceased donor kidney transplantation. In 2018, our center modified its protocol for stent removal after renal transplantation to the following:

1. Routine stent removal: within 2 weeks after transplant for living donor kidney transplantation and in 2–4 weeks for deceased donor kidney transplantation
2. Urgent stent removal: if a patient develops UTI, removal of the stent is sought once the infection is controlled
3. Emergent stent removal: unstable patients with severe sepsis due to UTI or fungal infection [21]

In our study, when the urinary stent was removed after 21 days of transplantation, there was a trend towards a higher odds of developing UTIs although this trend was not statistically significant (OR 1.43, *P*; 0.259, CI: 0.76–2.66). Of note, when the stent was removed on urgent basis not related to UTIs, such as a migrated or a fallen stent, there were statistically less UTIs. Nonetheless, the number of recipients who had urgent early removal of their stent was too low to yield conclusive statements. Our group had previously suggested that studying “UTIs related to urinary stent” defined as UTIs while the ureteral stent is still in vivo and up to two weeks after its removal is a better indicator of the impact of the timing of stent removal than reviewing UTIs for a total duration of 3 or 6 months after transplantation [29]. Multiple other studies have examined the impact of stent removal at different intervals form renal transplantation including at 4 weeks [30], 3 weeks [31], 2 weeks [32], one week [33], or 5 days [34] after renal transplantation. These studies have shown that early removal of ureteric stents following kidney transplantation may potentially reduce the incidence of UTI without a significant increase of major urological complications. Adoption of an earlier stent removal protocol may be beneficial.

The antibiotics prophylaxis protocol at our center used to consist of a combination of Bactrim DD every other day for six months along with norfloxacin 400 mg daily for the first 3 months. This protocol was based on the previous studies that evaluated the effects of dual prophylactic coverage with fluroquinolone and TMP/SXT versus TMP/SXT alone in post-kidney-transplant patients [35, 36]. This practice was modified in July 2018, and the current protocol consists of Bactrim SS for 6 months which is conjunctly used as prophylaxis for *Pneumocystis carinii* pneumonia [21, 37]. In this study, dual antibiotic prophylaxis (norfloxacin along with Bactrim DD) was not associated with the decreased risk of UTIs when compared to the standard prophylaxis with Bactrim SS daily.

The most common organisms in our study were *Escherichia coli* 51% and *Klebsiella* 18%. MDROs were very common and constitute about 42% of the microorganisms on the first episode and 50% on the second episode of UTI. Such high rates have also been reported in other studies [5, 12, 15, 17, 19, 23].

UTIs may have little or no impact on the long-term outcome of the graft survival [2, 12]. However, UTIs remain a leading cause of hospitalization after transplantation [3, 9] and the hospitalization rate due to UTIs was 49% of the first UTIs and 32% of the first recurrence in our study. Such high rate of hospitalization is associated with significant cost and can add to the burden of the healthcare system.

The limitation of this study is the retrospective nature of the study at a single center.

## 5. Conclusions

UTIs and recurrence are common in the first 6 months after renal transplantation. Age >40, female gender, neurogenic bladder, and transplantation abroad are associated with the increased risk of UTIs and recurrence.

In-hospital treatment is frequently required, and MDROs are common causative organisms.

Dual antibiotic coverage (when compared to Bactrim SS alone) was not associated with the decreased risk of UTI.

A controlled trial will be required to depict the best timing of stent removal after kidney transplantation.

## Figures and Tables

**Table 1:** Patients' characteristics.

Pt characteristics	Total	No UTI	UTI	*P*
	279	97	182	
Age	43.4 ± 15.8	42.1 ± 15.6	45.8 ± 16.0	0.063
Gender				
Male	165	128	37	<0.001
	59.10%	70.30%	38.10%	
Female	114	54	60	
	40.90%	29.70%	61.90%	

Diabetes mellitus	95	59	36	0.507
	34.10%	32.40%	37.10%	
Type I	25	19	6	0.099
	26.60%	32.80%	16.70%	
Type II	69	39	30	
	73.40%	67.20%	83.30%	

Donor type				
Deceased	55	34	21	0.636
	19.70%	18.70%	21.60%	
Living	224	148	76	
	80.30%	81.30%	78.40%	

Transplant abroad	21	9	12	0.032
	7.50%	4.90%	12.40%	

Pretransplant residual urine volume				
24-hour urine volume				
Anuria	68	45	23	0.805
	24.40%	24.70%	23.70%	
Oliguria	41	29	12	
	14.70%	15.90%	12.40%	
Normal	62	38	24	
	22.20%	20.90%	24.70%	

Preexisting urological abnormalities				
Treated urethral stricture	11	9	2	0.340
	3.90%	4.90%	2.10%	
Vesicoureteral reflux ( VUR)	7	3	4	0.242
	2.50%	1.60%	4.10%	
Neurogenic bladder	10	3	7	0.023
	3.60%	1.60%	7.20%	

Foley catheter				
Foley catheter removal time	5.8 ± 2.0	5.7 ± 1.9	6.1 ± 2.3	0.120
Foley catheter reinsertion	6	3	3	0.668
	2.20%	1.70%	3.10%	

Serum creatinine in mmol/L^*∗*^				
At 1 month	103.7 ± 47.2	105.1 ± 49.1	101.0 ± 43.5	0.493
At 6 months	99.0 ± 34.0	100.4 ± 34.9	96.4 ± 32.4	0.352

Immunosuppression status				
Induction therapy				
Basiliximab	107	72	35	0.607
	38.40%	39.60%	36.10%	
Thymoglobulin (ATG)	172	110	62	
	61.60%	60.40%	63.90%	
Rejection within 6 months	22	14	8	1
	7.90%	7.70%	8.20%	
Detected polyoma viremia	19	14	5	0.467
	6.80%	7.70%	5.20%	

UTI antibiotic prophylaxis				
TMP/SMX double strength + norfloxacin	70	49	21	0.385
	25.10%	26.90%	21.60%	
TMP/SMX single strength	209	133	76	
	74.90%	73.10%	78.40%	

Stent removal time (days)				
Stent removal time (days)	35.3 ± 28.0	34.2 ± 28.7	37.3 ± 26.8	0.376

Urgent stent removal due to non-UTI reasons				
Stent migration to urethra	6	5	1	<0.011
	2.1%	1.8%	0.30%	

Postrenal transplant surgical complications				
Urine leak	3	1	2	0.554
	1.10%	0.50%	2.10%	
Ureteral stenosis	6	3	3	0.668
	2.20%	1.60%	3.10%	

Continuous data were reported as (mean ± SD)^*∗*^, and categorical data were reported as numbers and percentages. ^†^Detected polyoma viremia >50 copies/ml.

**Table 2:** Timing and symptomatology and treatment of recurrent UTI after renal transplant.

*N*	1st UTI	2nd UTI	3ed UTI	4th UTI
	97	56	27	14
	–	57%	27%	14%
Days form transplant	40.8 ± 44.5	70.1 ± 45.4	89.9 ± 39.9	124.2 ± 44.5

Asymptomatic UTI	58	34	13	7
	63.7%	60.7%	48.1%	50.0%
Simple UTI	14	5	5	2
	14.4%	8.9%	18.5%	14.3%
Complicated UTI	12	8	2	4
	12.4%	14.3%	7.4%	28.6%
Complicated with bacteremia	6	5	4	1
	6.2%	8.9%	14.8%	7.1%

Multidrug-resistant organism (MDRO)	41	28	17	8
	42.3%	50.0%	63.0%	57.1%

Not treated	13	9	2	3
	13.4%	16.1%	7.4%	21.4%
Treated out-patient	36	29	11	6
	37.1%	51.8%	40.7%	42.9%
Treated in-patient	48	18	14	5
	49.5%	32.1%	51.9%	35.7%

**Table 3:** Predictors of first UTI after renal transplantation.

	*P* value	OR	95% CI	
Age <=40 years	Reference			
>40 years	0.012	2.176	1.187	3.986

Gender: male	Reference			
Female	<0.001	5.008	2.74	9.156

No DM	Reference			
DM type I	0.551	0.719	0.243	2.128
DM type II	0.053	1.948	0.99	3.833

Preemptive transplant	0.28	1.871	0.6	5.834
Transplant abroad	<0.001	5.008	2.607	27.05

Living donor	Reference			
Deceased donor	0.394	0.714	0.329	1.55

Basiliximab	Reference			
Thymoglobulin (ATG)	0.476	1.274	0.655	2.477

Neurogenic bladder	0.048	5.646	1.016	31.379
Vesicoureteral reflux	0.375	2.419	0.344	17.012

Stent removed <=21 days	Reference			
>21 days	0.259	1.43	0.768	2.663

Rejection	0.97	0.98	0.333	2.882
BK^†^	0.656	0.757	0.222	2.581
Foley >7 days	0.079	1.918	0.926	3.97

Bactrim SS^▲^	Reference			
Bactrim DS + *N* ^∗^	0.138	0.584	0.287	1.189

^▲^Trimethoprim/sulfamethoxazole 80/400 mg daily; ^∗^trimethoprim/sulfamethoxazole, 160/800 mg every other day + nitrofurantoin 400 mg daily. ^†^Detected polyoma viremia > 50 copies/ml.

## Data Availability

The data used to support the findings of this study are included within the article.

## Abbreviations

MDRO: Multidrug-resistant organism
Bactrim DS: Trimethoprim/sulfamethoxazole 160/800 mg
Bactrim SS: Trimethoprim/sulfamethoxazole 80/400 mg
UTI: Urinary tract infection
VUR: Vesicoureteral reflux.
